# Supporting Homework Compliance in Cognitive Behavioural Therapy: Essential Features of Mobile Apps

**DOI:** 10.2196/mental.5283

**Published:** 2017-06-08

**Authors:** Wei Tang, David Kreindler

**Affiliations:** ^1^ Discipline of Psychiatry Department of Medicine Memorial University of Newfoundland St. John's, NL Canada; ^2^ Division of Youth Psychiatry Department of Psychiatry Sunnybrook Health Sciences Centre Toronto, ON Canada; ^3^ Centre for Mobile Computing in Mental Health Department of Psychiatry Sunnybrook Health Sciences Centre Toronto, ON Canada; ^4^ Department of Psychiatry University of Toronto Toronto, ON Canada

**Keywords:** cognitive behavioral therapy, homework compliance, mobile apps

## Abstract

Cognitive behavioral therapy (CBT) is one of the most effective psychotherapy modalities used to treat depression and anxiety disorders. Homework is an integral component of CBT, but homework compliance in CBT remains problematic in real-life practice. The popularization of the mobile phone with app capabilities (smartphone) presents a unique opportunity to enhance CBT homework compliance; however, there are no guidelines for designing mobile phone apps created for this purpose. Existing literature suggests 6 essential features of an optimal mobile app for maximizing CBT homework compliance: (1) therapy congruency, (2) fostering learning, (3) guiding therapy, (4) connection building, (5) emphasis on completion, and (6) population specificity. We expect that a well-designed mobile app incorporating these features should result in improved homework compliance and better outcomes for its users.

## Homework Non-Compliance in CBT

Cognitive behavioral therapy (CBT) is an evidence-based psychotherapy that has gained significant acceptance and influence in the treatment of depressive and anxiety disorders and is recommended as a first-line treatment for both of these [1,2]. It has also been shown to be as effective as medications in the treatment of a number of psychiatric illnesses [3-6]. Homework is an important component of CBT; in the context of CBT, homework can be defined as “specific, structured, therapeutic activities that are routinely discussed in session, to be completed between sessions” [7]. Completion of homework assignments was emphasized in the conception of CBT by its creator, Aaron Beck [8]. Many types of homework are prescribed by CBT practitioners, including symptom logs, self-reflective journals, and specific structured activities like exposure and response prevention for obsessions and compulsions. These can be divided into the following 3 main categories: (1) psychoeducational homework, (2) self-assessment homework, and (3) modality-specific homework. Psychoeducation is an important component in the early stage of therapy. Reading materials are usually provided to educate the client on the symptomatology of the diagnosed illness, its etiology, as well as other treatment-relevant information. Self-assessment strategies, including monitoring one’s mood using thought records, teach the patients to recognize the interconnection between one’s feelings, thoughts, and behaviors [8]. For example, depressed patients may be asked to identify thinking errors in daily life and document the negative influences these maladaptive thinking patterns can produce on their behaviors. Various psychiatric disorders may require different types of modality-specific homework. For example, exposure to images of spiders is a treatment method specific to arachnophobia, an example of a “specific phobia” in the Diagnostic and Statistical Manual of Mental Disorders, 5th Edition (DSM-5) [9]. Homework is strategically created by the therapist to correct and lessen the patient’s psychopathology. The purpose of these exercises is to allow the patients to practice and reinforce the skills learned in therapy sessions in real life.

Homework non-compliance is one of the top cited reasons for therapy failure in CBT [10] and has remained a persistent problem in the clinical practice. Surveys of practitioners have suggested rates of non-adherence in adult clients of approximately 20% to 50% [10,11] while adherence rates in adolescents have been reported to be approximately 50% [12]. Many barriers to homework compliance have been identified in the literature; to facilitate discussions, they can be divided into internal and external factors. Internal factors originate from a client’s own psychological environment while external ones are created by external influences. Internal factors that have been identified include lack of motivation to change the situation when experiencing negative feelings, the inability to identify automatic thoughts, disregard for the importance or relevance of the homework, and the need to see immediate results [12-14]. Various external factors have also been identified, including the effort associated with pen-and-paper homework formats, the inconvenience of completing homework because of the amount of time consumed, not understanding of the purpose of the homework, lack of instruction, and failure to anticipate potential difficulties in completing the homework [14-16]. There is strong evidence suggesting that homework compliance is integral to the efficacy of CBT in a variety of psychiatric illnesses. In the treatment of depression with CBT, homework compliance has been correlated with significant clinical improvement and shown to predict decreases in both subjective and objective measures of depressive symptoms [17-23]. Similarly, homework compliance is correlated with short-term and long-term improvement of symptoms in anxiety disorders, including generalized anxiety disorder (GAD), social anxiety disorder (SAD), hoarding, panic disorder, and post-traumatic stress disorder (PTSD) [17,24-32]. Fewer studies have been done on homework compliance in other psychiatric conditions, but better homework compliance has been correlated with significant reductions in pathological behaviors in psychotic disorders [33,34], cocaine dependence [35,36], and smoking [37]. Two meta-analyses further support the notion that greater homework adherence is associated with better treatment outcomes in depression, anxiety-related disorders, and substance use [38,39].

## The Utility of Technology in Enhancing CBT Homework

Despite its demonstrated efficacy, access to CBT (as well as other forms of psychotherapy) remains difficult due to the limited number of practicing psychotherapists and the cost of therapy sessions [40]. With the rise of mass-market mobile communication devices such as the iPhone or other kinds of mobile devices with app capabilities (smartphones), new solutions are being sought that will use these devices to provide therapy to patients in a more cost-effective manner. Mobile phones with app capabilities are portable devices that combine features of a cellphone and a hand-held computer with the ability to wirelessly access the Internet. Over time, ownership of mobile phones in North America has grown [41,42] and progressively lower prices have further reduced barriers to their use and ownership [43,44]. As more and more people acquire mobile phones, the acceptance of and the demand for mobile health solutions have been on the rise [45]. Boschen (2008), in a review predating the popularization of the modern mobile phone, identified the unique features of the mobile telephone that made it a potentially suitable vehicle for adjunctive therapeutic applications: portability, acceptability, low initial cost, low maintenance cost, social penetration and ubiquity, “always on,” “always connected,” programmability, audio and video output, keypad and audio input, user-friendliness, and ease of use [46]. Over the last decade, modern mobile phones have supplanted the previous generation of mobile telephones; progressive increases in their computing power, ongoing advances in the software that they run and interact with (eg, JAVA, HTML5, etc.), common feature sets across different operating systems such as Google Inc.'s Android or Apple Inc.'s iOS, and adoption of common hardware elements across manufacturers (eg, touch screens, high-resolution cameras, etc) have enabled the development of platform-independent apps for mobile phones, or at least apps on different platforms with comparable functionality (eg, apps written for Apple's HealthKit or the apps written for Microsoft's HealthVault).

The popularization of the smartphone presents a unique opportunity to enhance CBT homework compliance using adjunctive therapeutic applications such that well-designed mobile software may be able to diminish barriers to CBT [40] by making CBT therapists' work more cost-effective. However, there are no guidelines and no existing research that directly address the design of mobile phone apps for this purpose. Given this gap in the literature, we searched MEDLINE (1946 to April 2015) and PsycINFO (1806 to April 2015) for all articles related to “cognitive behavioral therapy”, “homework”, “mobile applications” and “treatment compliance or adherence”, and reviewed articles related to (1) mobile technologies that address homework completion, (2) essential features of therapy, or (3) barriers to homework completion in CBT. In this article, we propose a collection of essential features for mobile phone-based apps that will optimally support homework compliance in CBT.

## A Proposed List of Essential Features for Mobile Apps That Optimally Support CBT Homework Compliance

In order to be effective for patients and acceptable to therapists, an optimal mobile phone app to support CBT homework compliance should conform to the CBT model of homework while addressing barriers to homework compliance. Tompkins (2002) provides a comprehensive guideline on the appropriate ways to provide CBT homework such that homework should be meaningful, relevant to the central goals of therapy, salient to focus of the session, agreeable to both therapist and client, appropriate to sociocultural context, practiced in session to improve skill, doable, begin small, have a clear rationale, include written instructions, and include a backup plan with homework obstacles [47]. In addition, the therapist providing the homework needs to be curious, collaborative, reinforce all pro-homework behavior and successful homework completion, and emphasize completion over outcome [47]. By combining Tompkins' guidelines with the need to reduce barriers to homework compliance (as described above), we obtained the following list of 6 essential features that should be incorporated into mobile apps to maximize homework compliance: (1) congruency to therapy, (2) fostering learning, (3) guiding therapy, (4) building connections, (5) emphasizing completion, and (6) population specificity.

### Congruency to Therapy

Any intervention in therapy needs to be relevant to the central goals of the therapy and salient to the focus of the therapeutic session. A mobile app is no exception; apps have to deliver useful content and be congruent to the therapy being delivered. There are different types of homework in CBT, including (1) psychoeducational homework; (2) self-assessment homework; and (3) modality-specific homework. Which types are assigned will depend on the nature of the illness being treated, the stage of treatment, and the specific target [48]. An effective app supporting homework compliance will need to be able to adjust its focus as the therapy progresses. Self-monitoring and psychoeducation are major components in the early stage of therapy. Thought records can be used in depression and anxiety while other disorders may require more specific tasks, such as initiating conversation with strangers in the treatment of SAD. Therefore, the treatment modules delivered via mobile phones should meet the specific needs of therapy at each stage of therapy, while also providing psychoeducation resources and self-monitoring capabilities.

#### Psychoeducational Homework

While there are large amounts of health-related information on the Internet, the majority of information is not easily accessible to the users [49]. Mobile apps can enhance psychoeducation by delivering clear and concise psychoeducational information linked to the topics being covered in therapy. As psychoeducation is seen as a major component of mobile intervention [50], it has been incorporated into several mobile apps, some of which have been shown to be efficacious in treating various psychiatric conditions, including stress [51], anxiety and depression [52], eating disorders [53], PTSD [54], and obsessive compulsive disorder (OCD) [55]. For example, Mayo Clinic Anxiety Coach is a mobile phone app “designed to deliver CBT for anxiety disorders, including OCD” [55]. The app contains a psychoeducational module that teaches the user on “the use of the application, the cognitive-behavioral conceptualization of anxiety, descriptions of each anxiety disorder, explanations of CBT, and guidance for assessing other forms of treatment” [55]. The benefits of delivering psychoeducation via a mobile phone app are obvious: the psychoeducational information becomes portable and is easily accessed by the patient. Furthermore, the information is also curated and validated by proper healthcare authorities, which builds trust and reduces the potential for misinformation that can result from patient-directed Internet searches. However, psychoeducation on its own is not optimal. Mobile interventions that also incorporate symptom-tracking and self-help interventions have resulted in greater improvement when used for depression and anxiety symptoms than those that deliver only online psychoeducation [50].

#### Self-Assessment Homework

In contrast to conventional, paper-based homework, mobile apps can support in-the-moment self-assessments by prompting the user to record self-report data about the user’s current state [56]. While information collected retrospectively using paper records can be adversely affected by recall biases [57], mobile apps enable the patient to document his or her thoughts and feelings as they occur, resulting in increased accuracy of the data [58]. Such self-assessment features are found in many mobile apps that have been shown to significantly improve symptoms in chronic pain [59,60], eating disorders [61], GAD [62], and OCD [55]. Continuing with the previous example, the Mayo Clinic Anxiety Coach offers a self-assessment module that “measures the frequency of anxiety symptoms” with a self-report Likert-type scale [55]. The app tracks users’ progress over time based on the self-assessment data; users reported liking the record of daily symptom severity scores that the application provides.

#### Modality-Specific Homework

Evidence suggests that a variety of modality-specific homework assignments on mobile apps are effective, including relaxation practices, cognitive therapy, imaginal exposure in GAD and PTSD [54,57], multimedia solutions for skill learning and problem solving in children with disruptive behavior or anxiety disorders [63], relaxation and cognitive therapy in GAD [62], or self-monitoring via text messages (short message service, SMS) to therapists in bulimia nervosa [61]. Mayo Clinic Anxiety Coach, for example, has a treatment module for OCD that “guides patients through the use of exposure therapy” [55]; patients can use this to build their own fear hierarchies according to their unique diagnoses. Users reported liking the app because it contains modality-specific homework that can be tailored to their own needs. Novel formats, such as virtual reality apps to create immersive environments, have been experimented with as a tool for facilitating exposure in the treatment of anxiety disorders with mostly positive feedback [64-66]. Apps that provide elements of biofeedback (such as heart rate monitoring via colorimetry of users' faces using the mobile phone's camera), have recently begun to be deployed. So-called ”serious games,“ (ie, games developed for treatment purposes), are also showing promise in symptom improvement in certain cases [51,67,68].

### Fostering Learning

Doing CBT homework properly requires time and effort. As noted above, any sense of inconvenience while doing the homework may hamper a patient’s motivation to complete the homework. While patients may appreciate the importance of doing homework, they often find the length of time spent and the lack of clear instructions discouraging, resulting in poor engagement rates [49,52]. Therefore, it makes sense that the tasks should be simple, short in duration to begin with, and include detailed instructions [47], since homework completion rates have been shown to be correlated with patients’ knowing exactly what to do [33,69]. Many apps incorporate text messaging-based services or personalized feedback to encourage dynamic interactions between the therapist and the client [59]. However, the types of homework delivered by these apps are fixed. An app that adapts the contents to the user’s progress in learning homework tasks would be more engaging and effective since therapy should be a flexible process by nature. Ideally, the app would monitor and analyze the user’s progress and adjust the homework's content and difficulty level accordingly. While the effectiveness of this type of app has not been studied, a similar app has been described in the literature for treating GAD [62]. This app, used in conjunction with group CBT, collected regular symptom rating self-reports from patients to track anxiety. Based on patients’ ratings, the app would respond with encouraging comments and invite patients to practice relaxation techniques or prompt the patient to complete specific built-in cognitive therapy modules if their anxiety exceeded a threshold rating. Despite the simple algorithm used to trigger interventions, use of the app with group CBT was found to be superior to group CBT alone.

### Guiding Therapy

Therapists have a number of important roles to play in guiding and motivating clients to complete homework. First, the therapist needs to address the rationale of the prescribed homework and work with the client in the development of the treatment plan [47]. Failure to do this has been identified as a barrier to homework compliance. Second, the therapist should allow the patient to practice the homework tasks during the therapy sessions [47] in order to build confidence and minimize internal barriers, such as the failing to identify automatic thoughts. Lastly, the therapist has to be collaborative, regularly reviewing homework progress and troubleshooting with the patients [47,70]; this can be done during or in between homework assignments, either in-person or remotely (ie, via voice or text messaging) [60,71].

Reviewing and troubleshooting homework has been seen as a natural opportunity for apps to augment the role of therapists. Individualized guidance and feedback on homework is found in many Internet-based or mobile apps that have been shown to be effective in treating conditions such as PTSD [72], OCD [55], chronic pain [59,60], depression and suicide ideation [71], and situational stress [73]. Moreover, providing a rationale for homework, ensuring understanding of homework tasks, reviewing homework, and troubleshooting with a therapist have each individually been identified as predictors of homework compliance in CBT [74,75]. However, despite incorporating a variety of features including self-monitoring, psychoeducation, scheduled reminders, and graphical feedback [52], automated apps with minimal therapist guidance have demonstrated elevated homework non-completion rates of up to 40%, which is less than ideal.

### Building Connections

The effects of technology should not interfere with but rather encourage a patient’s ability to build meaningful connections with others [76]. The therapeutic alliance between the therapist and the client is the strongest predictor of therapeutic outcome [77] and has been suggested to predict level of homework compliance as well [78]. While there is no evidence so far to suggest that technology-based interventions have an adverse effect on the therapeutic alliance [79,80], this conclusion should not be generalized to novel technologies as their impact on therapeutic alliance has not been well studied [81].

An arguably more significant innovation attributable to technology has been its potential to allow patients to form online communities, which have been identified as useful for stigma reduction and constructive peer support systems [82]. Online or virtual communities provide patients with a greater ability to connect with others in similar situations or with similar conditions than would be possible physically. Internet-delivered CBT that includes a moderated discussion forum has been shown to significantly improve depression symptoms [83]. Furthermore, professional moderation of online communities increases users’ trust of the service [84]. Therefore, including social platforms and online forums in a mobile app may provide additional advantages over conventional approaches by allowing easier access to social support, fostering collaboration when completing homework, and enabling communication with therapists.

### Emphasizing Completion

A patient’s need to see immediate symptomatic improvement is an impediment to homework compliance since the perception of slow progress can be discouraging to the user [35]. To address this issue, it is important for both therapists and mobile apps to emphasize homework completion over outcome [47]. While a therapist can urge the client to finish uncompleted homework during the therapy session to reinforce its importance [47,85], there is little a therapist can do in between therapy sessions to remind clients to complete homework. In contrast, a mobile app can, for example, provide ongoing graphical feedback on progress between sessions to motivate users [52,86], or employ automatic text message reminders, which have been demonstrated to significantly improve treatment adherence in medical illnesses [87]. These features have previously been incorporated into some technology-based apps for homework adherence when treating stress, depression, anxiety, and PTSD [52,54,88] with significant symptom improvement reported in one paper [71].

### Population Specificity

Homework apps should, where relevant or useful, explicitly be designed taking into account the specific characteristics of its target audience, including culture, gender, literacy, or educational levels (including learning or cognitive disabilities). One example of how culture-specific design features can be incorporated can be found in Journal to the West, a mobile app for stress management designed for the Chinese international students in the United States, which incorporates cultural features into its game design [89]. In this game, breathing activity is associated with the concept of “Qi” (natural energy) in accordance with Chinese traditions; the name of the game itself references to a famous Chinese novel and the gaming environment features inkwash and watercolor schemes of the East Asian style, making the experience feel more “natural” as reported by the users. A different approach to tailoring design is taken by the computer-based games described by Kiluk et al [68] that combine CBT techniques and multi-touch interface to teach the concepts of social collaboration and conversation to children with autism spectrum disorders. In these games, the touch screen surface offers simulated activities where children who have difficulties with peer engagement can collaborate to accomplish tasks. Children in this study demonstrated improvement in the ability to provide social solutions and better understanding of the concepts of collaboration. Although the population-specific design is intuitively appealing, the degree to which it can enhance homework compliance has yet to be investigated.

## Other Considerations

There are several additional issues specific to mobile apps that should be carefully considered when developing mobile apps for homework compliance. Because of screen sizes, input modes, the nature of electronic media, etc, standard CBT homework may need to be translated or modified to convert it into a format optimal for delivery via a mobile phone [47]. The inclusion of text messaging features remains controversial, in part because of concerns about client-therapist boundary issues outside the therapy sessions [90]. One potential solution is to use automated text messaging services to replace direct communication between the therapist and the client so the therapist can't be bombarded by abusive messages [52,61,91,92]. Privacy and security issues are also real concerns for the users of technology [93], although no privacy breaches related to text messaging or data security have been reported in studies on mobile apps so far [88,94-98]. Designers of mobile apps should ensure that any sensitive health-related or personal data is stored securely, whether on the mobile device or on a server.

Finally, while this paper focused on “essential” features of apps, this should not be misunderstood as an attempt to itemize all elements necessary for designing a successful piece of software. Good software design depends on many important elements that are beyond the scope of this paper, such as a well-designed user interface [99] that is cognitively efficient relative to its intended purpose [100] and which makes effective use of underlying hardware.

## Discussion

The popularization and proliferation of the mobile phone presents a distinct opportunity to enhance the success rate of CBT by addressing the pervasive issue of poor homework compliance. A variety of barriers exist in traditional, paper-based CBT homework that can significantly hamper clients’ motivation to complete homework as directed. The 6 essential features identified in this paper can each potentially enhance homework compliance. Therapy congruency focuses the features of the app on the central goal of therapy and fostering learning eases engagement in therapy by reducing barriers. Apps should help the therapist guide the client through therapy and not hinder the therapeutic process or interfere with patient’s building connections with others. It is crucial that homework completion be emphasized by the app, not just homework attempting. Population-specific issues should also be considered depending on the characteristics of targeted users.

As an example of how this applies in practice, “Mental Health Telemetry-Anxiety Disorders” (MHT-ANX) is a new mobile app developed by the Centre for Mobile Computing in Mental Health at Sunnybrook Health Sciences Centre in Toronto that helps patients monitor their anxiety symptoms using longitudinal self-report. The symptom log is therapy congruent to the practice of CBT since it promotes patients' awareness of their anxiety symptoms and the symptoms’ intensity. The simplicity of the app makes it easy for patients to learn to use, consistent with the need for fostering learning and increasing compliance. The MHT-ANX app was designed to share patient data with their clinicians, helping clinicians guide patients through therapy and more readily engage in discussion about symptom records, thus potentially enhancing the therapeutic relationship. Homework completion is emphasized both by automated text message reminders that the system sends and by questions presented by MHT-ANX that focus on how homework was done. While there are few population-specific design issues obvious at first glance in MHT-ANX, the focus groups conducted as part of our design process highlighted that our target group preferred greater privacy in our app rather than ease of sharing results via social media, and prioritized ease-of-use. While not yet formally assessed, reports from staff and early users suggest that MHT-ANX has been helpful for some patients with promoting homework compliance.

### Limitations and Future Challenges

The feature list we have compiled is grounded in current technology; as technology evolves, this list may need to be revised. For example, as artificial intelligence [101] or emotional sensing [102] develops further, we would expect that software should be able to dynamically modify its approach to the user in response to users' evolving emotional states.

### Conclusion

This paper presents our opinion on this topic, supported by a survey of associated literature. Our original intention was to write a review of the literature on essential features of apps supporting CBT homework compliance, but there was no literature to review. The essential features that are the focus of this article are summaries of key characteristics of mobile apps that are thought to improve homework compliance in CBT, but randomized trials assessing the impact of these apps on homework compliance have not yet been done. We would anticipate synergistic effects when homework-compliance apps are used in CBT (eg, if measures of progress collected from an app were used as feedback during therapy sessions to enhance motivation for doing further CBT work), but the actual impact and efficacy of therapy-oriented mobile apps cannot be predicted without proper investigation.

## Abbreviations

CBT: cognitive behavioral therapy
GAD: generalized anxiety disorder
MHT-ANX: Mental Health Telemetry-Anxiety Disorders
OCD: obsessive compulsive disorder
PTSD: post-traumatic stress disorder
SAD: social anxiety disorder
